# Establishing a Multi-Society Generative AI Task Force Within Radiology

**DOI:** 10.7759/cureus.64475

**Published:** 2024-07-13

**Authors:** Sabrina K Hsiao, Rachel M Treat, Ramin Javan

**Affiliations:** 1 Department of Radiology, George Washington University School of Medicine and Health Sciences, Washington, USA

**Keywords:** medical education technology, general radiology, radiology ai, chatgpt4, chatbot, task force, education, artificial intelligence, technology, health policy

## Abstract

Traditional artificial intelligence (AI) tools have already been implemented in clinical radiology for lesion detection and decision-making. Generative AI (GenAI), comparingly, is a new subset of machine learning that functions based on data probabilities to create content, offering numerous capabilities yet also uncertainties. Multidisciplinary collaboration is essential in safely harnessing the power of GenAI as it transforms medicine. This paper proposes creating a GenAI task force among radiological societies, including the American College of Radiology (ACR), Society of Imaging Informatics in Medicine (SIIM), Radiological Society of North America (RSNA), European Society of Radiology (ESR), Association of University Radiologists (AUR), and American Roentgen Ray Society (ARRS) for its integration into clinical care, health policy, and education. In this paper, we explore how a task force with guidelines will help radiologists and trainees develop essential strategies for integrating evolving AI-related technologies into clinical practice.

## Editorial

There has been significant research in the field of generative AI (GenAI) in medicine, as evidenced by over 3500 publications in PubMed at the time of this submission within the past year and a half since the release of ChatGPT (based on the search of the term "ChatGPT" on PubMed). ChatGPT has already been shown to improve radiological report quality and provide greater efficiency in workflow for creating summaries, patient-centered reports, and recommendations [1]. Other software such as Rad AI Omni can also generate report impressions based on the text in the current report. GenAI evidently improves efficiency, reduces omissions from the conclusion, and reduces radiologists' cognitive load, especially regarding interruptions to the image interpretation workflow [2]. With the field of radiology positioned at the forefront of artificial intelligence (AI) research and implementation, during the COVID-19 pandemic, the American College of Radiology’s (ACR) Data Science Institute (DSI) initiated an AI Community to bring together imaging societies, organizations, and federal agencies for public health. The ACR-DSI provides online data science services in collaboration with the Food and Drug Administration (FDA). These services utilize AI algorithms and patient scenarios to aid clinical management. Notably, the ACR-DSI integrates GenAI into its ACR AI LAB™, a toolkit for testing algorithms with patient scenarios, featuring sections for patients, radiologists, and referring providers. Users can then utilize a GPT-assisted tool to generate data from documentation, procedures, and reports. However, examples are limited, such as only three patient scenarios in the "For Radiologists" category [3]. Additional examples with instruction are warranted to advance GenAI use in clinical practice. 

To prevent misuse and unethical integration of AI algorithms into healthcare, the guidance of GenAI and AI through policy and regulation must occur. While the January 2024 multi-society statement on AI tools in radiology offers an extensive practical summary of issues in the development and implementation of AI tools in clinical practice, no specific mention of GenAI is made [4]. This new generation of publicly available AI tools not only encompasses but also a broader user group that includes patients, trainees, and clinicians, a wider set of use case applications are applicable. 

Generative artificial intelligence arms in radiology

Health Policy and Guidelines

AI development outpaces governance, presenting risks to safety and privacy. Proactive discussion about AI's role in healthcare, including relevant privacy and security implications, should drive policy-making about healthcare technologies and the protection of patient information. Policies should instill regulations about AI in healthcare to set safe standards, responsibility, and accountability on behalf of healthcare teams. For example, the “Safe, Secure, and Trustworthy Development and Use of Artificial Intelligence” offers federal guidance on the safe use of AI in multiple sectors, including healthcare and education [5]. A proposed task force for GenAI would similarly prioritize transparency, fairness, accountability, non-discrimination, and algorithm-auditing in medical decision-making.

Research and Clinical Applications

A task force could provide guidelines on scientific publishing and peer review for journals, for example, by offering online tools for authors and reviewers to screen manuscripts to ensure compliance with requirements, although the reliability of such tools may be difficult to establish. GenAI can also help streamline the Institutional Review Board application and grant writing processes, such as by assisting with administrative tasks and document preparation, suggesting approaches toward research funding requests, and scientific data validation. Furthermore, GenAI has shown promise in clinical settings, including summarizing reports, finding errors, enhancing patient education, data mining reports, radiomics, and protocoling [6]. Such high-stakes AI use would require government regulations and oversight, which the task force guidelines could address.

Education and Training

GenAI can offer a succinct yet comprehensive evaluation of medical school and residency applicants' experiences and motivations through its natural language processing capabilities. Committees should receive training and guidance on tools like ChatGPT for fair use and informed decision-making. Conversely, applicants can utilize GenAI to assist in their writing process, accessing templates, grammar checks, and idea organization, thereby leveling the playing field among applicants, including non-native English speakers. While currently lacking, an AI guide by the AUR could provide a user-friendly framework for applicants and admissions committees alike. Radiology training programs should allocate dedicated time for AI-specific training and offer a specific curriculum that includes GenAI. While the ACR-DSI offers AI training for clinical scenarios and has partnered with the American Academy of Ophthalmology for model testing, no partnerships with other specialty academies exist. A task force would encourage such collaboration to strengthen shared interests and offer Continuing Medical Education (CME).

Stakeholders

Patients

GenAI can revolutionize patient education. As patients access their medical records, they may have questions, seeking answers from physicians, but may instead refer to chatbots due to easy access and availability. While those can help patients understand complex medical jargon, risks of misinformation and hallucinations exist. Regardless, GenAI encourages patient autonomy and compliance, potentially enabling physicians to optimize time effectively. A task force could outline ways to provide personalized care with pre- and post-imaging/procedure recommendations when using interactive chatbots. Of note, the topic of patient consent in the setting of using patient data for training of GenAI tools as well as using such tools in the clinical settings should be discussed.

Radiologists and Trainees

GenAI tools can reduce radiologist workload and enhance workflow, extensively explored in the literature, ranging from structured report generation and summarization to assisting with decision support through streamlined protocoling and billing [6]. A task force taking into consideration guidelines from government agencies would provide direction in the safe, proper use of GenAI tools in clinical settings, including the use of AI-powered content/media generators. Academic radiologists, radiology applicants, residents, and fellows can also benefit from clearer rules, strategies, and guidelines pertaining to the use of large language models in educational settings and within admissions committees.

Clinicians

GenAI can support clinicians across specialties by providing decision support aligned with ACR appropriateness criteria while minimizing redundancy and patient radiation. It can aid in time-consuming tasks like insurance pre-authorizations, ensuring homogeneity in criteria provided to insurers, and enhancing successful coverage. Additionally, it can simplify and translate radiology reports, reducing confusion among non-radiological colleagues and facilitating guideline adoption across clinical specialties. These applications hold promise for reducing physician burnout and streamlining workflow through responsible and guided implementation.

Healthcare Organizations and Developers

The task force should ensure that GenAI tools used in healthcare organizations comply with Health Insurance Portability and Accountability Act (HIPAA) regulations. Caution is also warranted to prevent medical errors, as evidenced by a study in which ChatGPT4 generated responses to cancer patient inquiries, with 7% deemed unsafe by participating radiation oncologists if left unedited [7]. Before implementation of GenAI tools, integration into IT and clinical workflows should also undergo pilot testing to mitigate errors. Similarly, the task force must prioritize patient data protection against AI-related hacking threats, addressing the roles of open-source vs commercial software. GenAI interestingly could enhance cybersecurity by predicting and preventing threats, detecting anomalies, analyzing malware, and improving biometrics. Radiology organizations should address security measures with developers before implementation, and developers should disclose financial and intellectual property protection policies. Furthermore, developers should ensure AI's actions align with human values, create explainable AI-minimizing “black-box” algorithms, and offer transparency of data sets used for GenAI training.

Applications, Mission Statement, and Objectives

As an overview of the proposed GenAI task force, two comprehensive tables summarizing the task force's aims are listed below. The first table outlines the task force's potential applications for healthcare and educational organizations; the second table conveys the task force's mission statement and objectives (Tables 1, 2).

**Table 1:** Task force framework and considerations for implementing GenAI in radiology ACR: American College of Radiology; SIIM: Society for Imaging Informatics in Medicine; RSNA: Radiological Society of North America; ESR: European Society of Radiology; AUR: Association of University Radiologists; ARRS: American Roentgen Ray Society; CME: Continuing Medical Education; GenAI: generative artificial intelligence, HIPAA: Health Insurance Portability and Accountability Act; IT: information technology Table Credits: Sabrina Hsiao, Rachel Treat, and Ramin Javan

Generative artificial intelligence arms in radiology	
Health policy and guidelines (ACR/SIIM)	Protecting patient safety and privacy
	Public health and preventive imaging guidance
	Regulatory compliance and guideline publication
Research and clinical use (RSNA/ESR)	Scientific publishing, peer review, and grant writing
	Research funding and scientific validation
	Up-to-date technical reports
Admissions and education (AUR/ARRS)	Residency applicant guidance
	Admissions committee guidance
	Residency curriculum and CME development
Stakeholders and considerations	
Patients	Enhanced accessibility, compliance, and digestibility of information
	Mitigating misinformation, disinformation, and bias
	Personalized care and pre-/post- imaging/procedure recommendations
	Informed consent and ethical/legal implications
Radiologists and trainees	Improved efficiency, radiology decision support, and report generation
	Workflow optimization, protocoling, and billing
	Radiomics and data extraction
	Responsible use of AI-powered multimedia generation and interactive chatbots
Clinicians	Clinical decision support for ordering imaging exams and improved exam history
	Insurance pre-authorizations
	Radiology report simplification and translation
	Adoption of radiology guidelines in the clinical and specialty realms
Healthcare organizations	HIPAA compliance
	Cybersecurity
	Pilot testing to mitigate misuse and errors
	Seamless IT and clinical integration
Developers	Patient data protection and hacking/jailbreak prevention
	Financial disclosures and intellectual property protection
	Alignment, explainable AI, and training data transparency
	Autonomous agents

**Table 2:** GenAI task force overarching aims ACR: American College of Radiology; RSNA: Radiological Society of North America; AUR: Association of Academic Radiology; ARRS: American Roentgen Ray Society; GenAI: generative artificial intelligence, CME: Continuing Medical Education Table Credits: Sabrina Hsiao, Rachel Treat, and Ramin Javan

Generative artificial intelligence task force mission statement
The GenAI radiology task force will serve as a united coalition of stakeholders, including but not limited to leaders from ACR, RSNA, AUR, and ARRS, which will discuss the integration of GenAI into all facets of radiology including health policy, clinical care, and education, with the ultimate goal of providing proactive, thorough, trustworthy guidelines for its safe and efficacious implementation.
Generative artificial intelligence task force objectives
Evaluating GenAI models through collaborative fine-tuning and testing using shared, standardized medical datasets for verified accuracy and clinical utility.
Establishing best practices for developing GenAI tools, such as open-source frameworks and protocols for model training with medically accurate data.
Advising on policies around GenAI in patient care, ensuring transparency and accountability.
Multidisciplinary entities, including radiologists, other specialty doctors, developers, and governmental agencies, should collaborate to enhance public health and patient care.
Leading public and physician education through social media campaigns and CME courses on GenAI capabilities, limitations, and ethical use.
Encouraging underrepresented groups in GenAI programming through mentorships, research investment, and skills training.
Exploring the use of AI virtual assistants to augment radiologists’ workflow.

Ethical, structural, and global considerations 

Ethical Considerations

Though GenAI has numerous beneficial applications, its use poses ethical concerns. Using GenAI-trained systems requires patient data, and maintaining the anonymity of patient data remains a complex yet important consideration that may pose risk; furthermore, potential drawbacks include addressing insurance costs, having AI systems with limited context, and facing discrimination based on data-related decisions made [4]. Data used to train AI algorithms may lead to biased healthcare decisions, which can lead to AI systems potentially furthering prevalent disparities in healthcare outcomes among different demographic groups [8]. A GenAI task force would address these concerns by seeking training systems that include diverse, representative data from patients with varying degrees of access to healthcare technologies. GenAI task force members would have required modules on healthcare inequities and equitable outcomes to promote equitable standards of care. 

GenAI can also produce content independently, which may be inaccurate and misguide the reviewing process for scientific journals. For example, a recently withdrawn article inadvertently contained “…I am an AI language model” in its published text [9]. To ensure similar errors do not interfere with GenAI’s use in academia, the GenAI task force could offer step-by-step instructions on how to use GenAI for planning, grammar edits, and suggestions for concision. The GenAI task force would also emphasize that any GenAI-produced content must be proofread and include human input to avoid similar AI-generated errors. However, a challenge of GenAI use for scientific publication is clarifying how GenAI can comply with each user’s respective journal requirements. To address this, the GenAI task force could provide instructions on how to best use GenAI to comply with common radiological journal requirements. These instructions would be offered through an explicit Portable Document Format (PDF) and informational video. A GenAI task force email for specific questions would need to be made, as well as a website. A website would consolidate and display the task force mission statement, core objectives, and any PDF or video instructions on GenAI use.

Structural Plan

Creating a task force requires logistical and organizational considerations. First, there should be designated leaders with distinct duties. There should be a hierarchy with a president who guides meetings and oversees the members; a vice president who recruits members and helps closely with the president in oversight; a treasurer who records funding and contacts donors; and a secretary who handles emails, social media posts, and letters of correspondence. There would also need to be a team to the monitor progress of members using GenAI to ensure efficient, ethical use of systems. Designated tasks would need to be explicitly stated with each leader’s respective signature to ensure accountability and transparency. Furthermore, task force members would have specific objectives to follow and be required to attend meetings. Meetings would be held on a monthly basis.

Global Implications

From a global perspective, the GenAI task force should collaborate with existing AI task groups. For example, the World Health Organization and the International Telecommunication Union have established a Focus Group on AI for Health (FG-AI4H) with similar initiatives [10]. The Global Digital Health Partnership consists of over 30 countries and territories dedicated to defining best practices for digital health policy. If the GenAI task force could become affiliated with the FG-AI4H, it would ensure members advance relevant skill sets and stay updated on emerging technologies globally.

GenAI holds immense potential in transforming radiology and healthcare, requiring attention to patient privacy, regulatory compliance, and seamless integration into clinical workflows. Creating a task force, comprising key stakeholders, is crucial to ensure the safe and effective adoption of these new tools. By fostering multidisciplinary collaboration, setting standardized guidelines, and advocating for transparent and accountable practices, the task force would achieve its mission to provide proactive, thorough, and trustworthy guidelines. This initiative aims to maximize the benefits of GenAI through advancing patient care in radiology while protecting patient safety and ethical standards.
